# Predicting Fetal Growth with Curve Fitting and Machine Learning

**DOI:** 10.3390/bioengineering12070730

**Published:** 2025-07-03

**Authors:** Huan Zhang, Chuan-Sheng Hung, Chun-Hung Richard Lin, Hong-Ren Yu, You-Cheng Zheng, Cheng-Han Yu, Chih-Min Tsai, Ting-Hsin Huang

**Affiliations:** 1 Department of Computer Science and Engineering, National Sun Yat-sen University, Kaohsiung 804, Taiwan; hzhang@g-mail.nsysu.edu.tw (H.Z.); yczheng7652@gmail.com (Y.-C.Z.); yu.beck@g-mail.nsysu.edu.tw (C.-H.Y.); tcmnor@cgmh.org.tw (C.-M.T.); guys0510@gmail.com (T.-H.H.); 2 Department of Pediatrics, Chang Gung Memorial Hospital-Kaohsiung Medical Center, Kaohsiung 833, Taiwan; yuu2004taiwan@yahoo.com.tw; 3 Graduate Institute of Clinical Medical Sciences, College of Medicine, Chang Gung University, Taoyuan 333, Taiwan; 4 Division of Cardiology, Department of Internal Medicine, Kaohsiung Chang Gung Memorial Hospital, Kaohsiung 833, Taiwan

**Keywords:** curve fitting, machine learning, polynomial regression, fetal growth, prenatal ultrasound

## Abstract

Monitoring fetal growth throughout pregnancy is essential for early detection of developmental abnormalities. This study developed a Taiwan-specific fetal growth reference using a web-based data collection platform and polynomial regression modeling. We analyzed ultrasound data from 980 pregnant women, encompassing 8350 prenatal scans, to model six key fetal biometric parameters: abdominal circumference, crown–rump length, estimated fetal weight, head circumference, biparietal diameter, and femur length. Quadratic regression was selected based on a balance of performance and simplicity, with R^2^ values exceeding 0.95 for most parameters. Confidence intervals and real-time anomaly detection were implemented through the platform. The results demonstrate the potential for efficient, population-specific fetal growth monitoring in clinical settings.

## 1. Introduction

Ultrasound imaging plays a critical role in prenatal care by enabling accurate fetal monitoring and early detection of complications. As the most widely adopted prenatal examination method, ultrasound is favored for its portability, convenience, and absence of radiation exposure [1,2]. The American College of Obstetricians and Gynecologists (ACOG) recommends routine ultrasound scans for all pregnant women [3]. Compared to estimates based on the last menstrual period, early ultrasound provides more accurate gestational age assessment and facilitates the detection of clinically unsuspected anomalies, such as fetal malformations and multiple pregnancies. It is also commonly used to estimate fetal weight and evaluate whether the fetus is developing within the expected growth range for its gestational age [4,5]. Abnormal fetal weight—either macrosomia or growth restriction—is associated with increased risk of complications, including gestational diabetes and preeclampsia.

Recent advances in artificial intelligence have enhanced fetal weight estimation, even in cases lacking complete ultrasound data [6,7,8]. AI-based models have shown promising performance in generating reliable predictions using maternal and clinical variables [9,10,11,12].

However, despite these clinical benefits, the fetal growth references currently in use are predominantly derived from international populations, which may not accurately represent the genetic, lifestyle, and healthcare characteristics specific to the Taiwanese population. This disparity limits the precision and effectiveness of growth-based anomaly detection in local clinical settings. To address this limitation, we propose the development of a localized fetal growth reference curve grounded in real-world data collected from Taiwanese newborns [13,14,15]. Region-specific modeling has been shown to yield more accurate predictions compared to generalized datasets, as demonstrated in studies focused on the Japanese population [16].

To support this objective, we introduce a web-based data collection and visualization platform specifically designed for prenatal monitoring. A distinguishing feature of our platform is its ability to collect data from two biologically related generations mothers and their newborns a level of granularity rarely seen in international databases such as TriNetX. This infrastructure enables not only population-specific reference modeling but also intergenerational analysis of growth patterns.

Traditionally, pregnancy-related data collection has relied heavily on paper-based questionnaires and manual data entry, both of which are labor-intensive and susceptible to transcription errors, omissions, and delays in clinical decision-making. These limitations hinder real-time feedback to expectant mothers and reduce the utility of the data for both clinical care and research. Furthermore, the absence of centralized, structured databases has historically impeded timely and evidence-based updates to fetal growth references, especially for localized populations.

To overcome these challenges, we developed a secure, web-based platform that digitizes the input and visualization of maternal and fetal health parameters. The platform supports responsive web interfaces accessible via both mobile and desktop devices, thereby enhancing accessibility and user engagement. By incorporating HTTPS encryption and account-based access control, it ensures data privacy and complies with ethical standards in clinical research. Authorized researchers are further empowered to execute structured queries and download anonymized datasets, thereby extending the platform’s applicability to longitudinal studies and health policy development.

Building on this digital infrastructure, the present study integrates traditional curve-fitting methods [17] with supervised machine learning techniques to construct a robust, data-driven fetal growth model. Specifically, we employ polynomial regression—using linear, quadratic, and cubic functions—to model statistical relationships between gestational age and key biometric indicators such as fetal weight, head circumference, and abdominal circumference. Among these models, the quadratic function provided the best balance between computational efficiency and model performance, achieving high coefficients of determination (R^2^ > 0.95) across most metrics. The fitting process minimizes the sum of squared residuals via the least-squares method, while model selection is guided by comparative R^2^ values and 10-fold cross-validation to ensure robustness and generalizability.

To further support clinical decision-making, we incorporate a rule-based anomaly detection framework grounded in statistical confidence intervals. By calculating the standard deviation for each fitted parameter, the platform dynamically generates upper and lower bounds (±2σ) to delineate the 95% confidence interval. Biometric values falling outside this range are automatically flagged as potential outliers, indicating risks such as intrauterine growth restriction (IUGR) or macrosomia. These alerts enable timely clinical intervention and promote proactive prenatal care.

## 2. Related Works

Fetal growth monitoring has long been a central focus of prenatal healthcare and biomedical research. Numerous computational approaches have been developed to model fetal development and identify abnormal growth trajectories. The existing literature can be broadly categorized into three methodological directions: (1) statistical and population-based growth standards, (2) machine-learning-based fetal growth prediction, and (3) deep learning approaches for automated ultrasound image analysis.

### 2.1. Statistical and International Growth Standards

This section discusses the limitations of widely used international statistical fetal growth standards and highlights the need for localized models in clinical practice. One of the most influential initiatives in fetal growth standardization is the INTERGROWTH–21st Project, proposed by Papageorghiou et al. [18], which established international fetal growth reference curves using longitudinal ultrasound data from low-risk pregnancies in eight geographically diverse countries. While this framework provides a unified global standard, it assumes universal applicability and overlooks population-specific differences stemming from genetics, maternal nutrition, and disparities in healthcare systems.

To improve inclusivity, the World Health Organization (WHO) introduced fetal growth charts based on a broader multinational cohort [19]. Although this dataset introduced greater ethnic diversity, it still failed to adequately capture regional variations. For example, healthy Taiwanese fetuses often present with smaller abdominal circumference and estimated fetal weight values compared to their Western counterparts, even under normal gestational conditions.

In response to such disparities, Buck Louis et al. [20] conducted the NICHD Fetal Growth Studies, generating race and ethnicity-specific growth standards for African American, Hispanic, Asian, and Caucasian populations within the United States. However, comprehensive, population-specific fetal growth curves for Taiwanese populations are still lacking. This absence of localized standards limits the precision of anomaly detection in domestic clinical practice. Our study aims to address this gap by developing a region-specific reference model using real-world prenatal data collected from Taiwanese hospitals.

### 2.2. Machine-Learning-Based Fetal Growth Prediction

To move beyond traditional statistical methods, researchers have investigated supervised machine learning approaches for fetal growth prediction. Kuhle et al. [11] evaluated models such as logistic regression, decision trees, random forests, and support vector machines (SVMs) for identifying fetal growth abnormalities. Their findings demonstrated that machine learning models consistently outperformed classical regression techniques in terms of accuracy and sensitivity. Nevertheless, the lack of interpretability in many of these models poses a significant barrier to clinical adoption, where explainable outputs are essential.

To enhance clinical viability, Lu et al. [6] proposed an ensemble learning model using XGBoost to estimate fetal weight based solely on maternal demographic and clinical data. Their model achieved performance comparable to ultrasound-based methods and showed promise for deployment in resource-limited settings. However, it lacked interactive features and real-time evaluation capabilities, which are critical for clinical decision support systems.

Building on these findings, our work introduces a polynomial regression framework with ridge regularization to strike a balance between model transparency and predictive accuracy. This lightweight, mathematically interpretable model can be easily integrated into web-based platforms, enabling real-time anomaly detection based on ±2 standard deviation thresholds and providing actionable insights for both clinicians and patients. In addition, Lee et al. [14] proposed a machine learning model to predict late-onset fetal growth restriction (FGR), utilizing clinical parameters from routine prenatal checkups. Their work demonstrated that models such as XGBoost and logistic regression could achieve high predictive accuracy in real-world clinical settings, further supporting the practical feasibility of machine learning in fetal health monitoring.

### 2.3. Deep Learning for Automated Biometric Extraction

Recent advances in deep learning have significantly enhanced medical image analysis, including the automation of fetal biometric estimation from ultrasound images. Oghli et al. [21] developed a convolutional neural network (CNN) to directly predict head and abdominal circumference from ultrasound scans, achieving high accuracy and reducing inter-operator variability.

Van den Heuvel et al. [22] further extended this approach by incorporating attention mechanisms and multi-scale feature extraction to improve predictions of fetal weight and femur length, particularly under noisy or low-resolution conditions. Although these deep learning models demonstrated state-of-the-art performance, they require considerable computational resources, typically depend on GPU acceleration, and function as black boxes with limited interpretability—characteristics that limit their feasibility in routine clinical workflows.

In contrast, our proposed system prioritizes model interpretability and computational efficiency to support real-time clinical decision-making. By leveraging closed-form polynomial equations and analytically derived confidence intervals, the system facilitates reliable, on-demand evaluation without the need for high-performance computing infrastructure.

Despite considerable progress in fetal growth modeling, key gaps remain unresolved. Most existing growth standards—including those from INTERGROWTH-21st and WHO—are derived from multiethnic or Western populations and do not adequately reflect the physiological characteristics of East Asian, particularly Taiwanese, fetuses. Although the NICHD study introduced race-specific references for U.S. populations, no equivalent localized standard exists for Taiwan, reducing the relevance and accuracy of these models in domestic prenatal care.

Moreover, while modern machine learning and deep learning methods achieve high predictive accuracy, their lack of interpretability and high computational cost hinder their deployment in real-time clinical environments. Black-box neural networks, though powerful, offer limited transparency, making them less suitable for systems that require clinician trust and operational clarity.

To bridge these gaps, we propose an AI-enabled, web-based clinical platform underpinned by a localized dataset comprising 980 pregnancies and 8350 ultrasound examinations from Taiwanese hospitals. Our system employs a quadratic polynomial regression model with ridge regularization, offering a transparent and efficient mechanism for fetal growth prediction. The model is integrated into a responsive interface supporting real-time data input, visualization, and anomaly detection using ±2 standard deviation confidence intervals. Its robustness was validated through 10-fold cross-validation across six key biometric parameters. This study represents a pioneering step toward a clinically deployable, population-specific fetal growth monitoring system tailored to the Taiwanese healthcare landscape.

## 3. Methods

### 3.1. Participants

This study enrolled 980 pregnant women who received prenatal care at Kaohsiung Chang Gung Memorial Hospital. All participants provided written informed consent to contribute anonymized clinical data for research purposes. The dataset, jointly curated with National Sun Yat-sen University, was fully anonymized prior to analysis to remove all personally identifiable information. As such, no identity-related data were disclosed, and individual reconsent was not required. Given the rigorous anonymization protocols, the study qualifies for exemption from Institutional Review Board (IRB) review in accordance with prevailing ethical guidelines.

### 3.2. Data Collection Website

**Figure 1:**
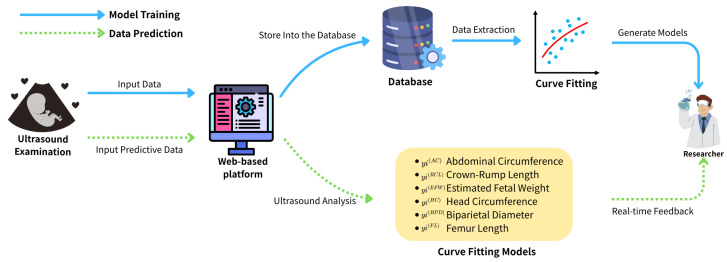
Schematic of the structure of this paper.

**Figure 2:**
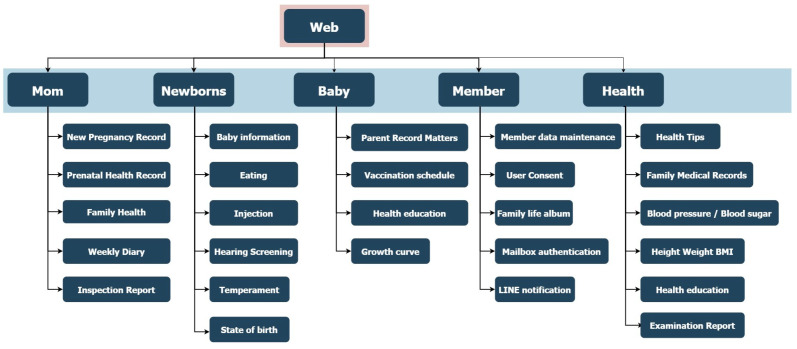
Sitemap of the data collection website.

The data collection platform was developed using the C# ASP.NET framework for backend implementation, ensuring scalability and integration with secure server-side operations. The front-end interface was designed with the BOOTSTRAP framework, enabling fully responsive webpages accessible via both mobile and desktop devices. The website is publicly available at https://idohad.net.nsysu.edu.tw (accessed on 29 June 2025), and its full functionality is illustrated in Figure 1 and Figure 2.

To ensure data security and user privacy, the platform employs HTTPS encryption, which safeguards all data transmissions against interception. In addition, the system features an account-based login mechanism to enforce access control and restrict unauthorized use, thereby maintaining compliance with standard clinical data protection protocols.

### 3.3. Data

This study compiled a dataset of essential biometric parameters derived from ultra-sound imaging obtained during routine prenatal examinations. The following six fetal growth indicators were recorded and analyzed:

1. Abdominal Circumference (AC): Reflects liver size and fat deposition, serving as a critical marker for estimating fetal weight and detecting intrauterine growth restriction (IUGR).
2. Crown–rump length (CRL): Measured in early gestation, it provides the most accurate estimation of gestational age during the first trimester.
3. Fetal Weight (EFW): Estimated through established biometric formulas, it is a central indicator for assessing fetal growth trajectory and potential macrosomia.
4. Head Circumference (HC): Indicates cranial and neurological development and is particularly important in assessing microcephaly or hydrocephalus.
5. Skull Diameter (Biparietal Diameter or BPD): A standard measurement for fetal brain development, often used alongside HC.
6. Thigh Length (Femur Length or FL): Represents long bone development and serves as a supplementary metric in estimating fetal age and weight.

These measurements collectively provide a multidimensional view of fetal morphometrics and are widely used in obstetric practice to assess whether fetal growth is proceeding within expected physiological ranges across gestational age.

To facilitate model development and performance evaluation, the dataset was randomly partitioned into training and testing subsets at a standard 70:30 ratio. This division ensures that model training captures sufficient data diversity, while testing allows unbiased evaluation of generalization ability. Each biometric parameter was subjected to polynomial curve fitting using three different models: linear (first-order), quadratic (second-order), and cubic (third-order) regression.

### 3.4. Data Analysis

Curve fitting is a foundational technique for modeling relationships between variables in structured datasets. In this study, we employed polynomial regression—specifically linear (degree 1), quadratic (degree 2), and cubic (degree 3) models—to approximate the relationship between gestational age and key fetal biometric parameters, including abdominal circumference, estimated fetal weight, head circumference, biparietal diameter, and femur length.

The selection of low-degree polynomial functions was motivated by the predominantly linear or quadratic growth patterns observed in fetal development. Since these biometric indicators generally increase with gestational age, polynomial models provide both theoretical relevance and computational efficiency. Compared to more complex methods such as neural networks, polynomial regression enables fast training and low-latency inference—advantages that are especially valuable for real-time clinical applications.

To mitigate overfitting and enhance generalizability, we adopted ridge regression, a regularized variant of least squares. The dataset was initially divided into training (70%) and testing (30%) subsets, and model performance was further assessed using 10-fold cross-validation. Among the three candidate models, the quadratic polynomial consistently offered the best balance between predictive accuracy and model simplicity.

The final fitted equations were integrated into a web-based clinical platform to enable real-time evaluation of fetal growth. As new biometric values are entered, the system instantly assesses their alignment with expected growth trajectories, facilitating early detection of anomalies such as intrauterine growth restriction or macrosomia at the point of care.

By combining interpretable statistical modeling with a practical digital interface, this approach delivers a robust and efficient framework for prenatal monitoring. The transparency and speed of polynomial regression make it particularly well suited for time-sensitive, resource-constrained clinical environments.

### 3.5. Curve Fitting Algorithm

To support the real-time clinical evaluation described above, we detail here the mathematical formulation of our polynomial regression approach used for fetal growth modeling. This section explains the algorithmic framework, parameter estimation process, and model evaluation metrics.

In this study, we employ a polynomial regression-based curve fitting method to model fetal biometric growth trends across gestational age. Given a dataset consisting of *n* observed pairs $\left(x_{i},y_{i}\right)$, where each $x_{i}\in R$ denotes the gestational age in days, and $y_{i}\in R$ represents the measured fetal biometric value (e.g., abdominal circumference and femur length), we seek to find a function f(x;θ) that accurately captures the underlying growth pattern. Table 1 summarizes the biometric variables used in the modeling process.

We define the predictive model as a polynomial function of degree *d*, which can be expressed compactly using basis functions. Let $\phi \left(x\right)={\left[1,x,x^{2},\ldots ,x^{d}\right]}^{T}$ be the vector of polynomial basis functions, and let $\theta ={\left[\theta_{0},\theta_{1},\ldots ,\theta_{d}\right]}^{T}$ denote the corresponding coefficient vector. Formula (1) is as follows:

(1) $$\begin{array}{c} f\left(x;\theta \right)=\phi {\left(x\right)}^{T}\theta =\sum_{k=0}^{d}\theta_{k}x^{k} \end{array}$$

To estimate the optimal parameters θ, we minimize the sum of squared errors between the predicted values and the observed outputs. Let $y={\left[y_{1},y_{2},\ldots ,y_{n}\right]}^{T}\in R^{n}$ be the vector of observed measurements and $\Phi \in R^{n\times (d+1)}$ be the design matrix whose *i*-th row is $\phi {\left(x_{i}\right)}^{T}$. Explicitly, these matrices can be expressed as Formula (2):

(2) $$\Phi =\left[\begin{array}{ccccc} 1 & x_{1} & x_{1}^{2} & \ldots & x_{1}^{d}\\ 1 & x_{2} & x_{2}^{2} & \ldots & x_{2}^{d}\\ \vdots & \vdots & \vdots & \ddots & \vdots \\ 1 & x_{n} & x_{n}^{2} & \ldots & x_{n}^{d} \end{array}\right],\;y=\left[\begin{array}{c} y_{1}\\ y_{2}\\ \vdots \\ y_{n} \end{array}\right]$$

The cost function to be minimized is then defined as

(3) $$\begin{array}{c} J\left(\theta \right)={∥y-\Phi \theta ∥}_{2}^{2}=\sum_{i=1}^{n}{\left(y_{i}-f\left(x_{i};\theta \right)\right)}^{2} \end{array}$$

Taking the gradient of J(θ) with respect to θ and setting it to zero yields the normal equation. Solving this yields the closed-form solution:

(4) $$\begin{array}{c} \theta^{*}={\left(\Phi^{T}\Phi \right)}^{-1}\Phi^{T}y \end{array}$$

Here, $\Phi^{T}\Phi$ is the Gram matrix of the design matrix Φ, and its invertibility assumes that the columns of Φ are linearly independent. In practice, when multicollinearity is present or when the number of basis functions is large relative to the number of data points, this matrix may become ill conditioned. To address this, we introduce ridge regression, a regularized version of least squares estimation. By adding a penalty term proportional to the squared $L^{2}$-norm of the parameter vector, the cost function becomes well posed. The ridge solution is given by

(5) $$\begin{array}{c} \theta_{\mathrm{ridge}}={\left(\Phi^{T}\Phi +\lambda I\right)}^{-1}\Phi^{T}y, \end{array}$$

where λ>0 is the regularization parameter that controls the trade-off between fitting the data and minimizing the model complexity, and I is the identity matrix of dimension (d+1)×(d+1). The purpose of λ>0 is to reduce overfitting and improve model stability, especially in cases of multicollinearity among polynomial basis terms. It prevents the coefficient estimates from becoming too large by penalizing their L2 norm. In our implementation, we selected λ>0 empirically based on preliminary cross-validation over a small range of values (e.g., 0.01, 0.1, 1, and 10) on the training data. The model showed low sensitivity to λ>0 due to the low polynomial order, and we chose λ>0 = 0.1 to ensure numerical stability while maintaining predictive performance.

Once the model is fitted, its performance is evaluated using the coefficient of determination $R^{2}$, which quantifies the proportion of variance in the target variable that is explained by the model. It is computed as

(6) $$\begin{array}{c} R^{2}=1-\frac{\sum_{i=1}^{n}{\left(y_{i}-{\hat{y}}_{i}\right)}^{2}}{\sum_{i=1}^{n}{\left(y_{i}-\bar{y}\right)}^{2}}, \end{array}$$

where ${\hat{y}}_{i}=f\left(x_{i};\theta \right)$ is the predicted value for the *i*-th input, and $\bar{y}=\frac{1}{n}\sum_{i=1}^{n}y_{i}$ is the mean of all observed values. Another common metric is the root mean squared error (RMSE), which measures the standard deviation of the residuals:

(7) $$\begin{array}{c} \mathrm{RMSE}=\sqrt{\frac{1}{n}\sum_{i=1}^{n}{\left(y_{i}-{\hat{y}}_{i}\right)}^{2}} \end{array}$$

These metrics allow us to compare models of different polynomial degrees and assess how well the model generalizes to unseen data. In this work, we evaluated polynomial models of degrees d=1 (linear), d=2 (quadratic), and d=3 (cubic). The quadratic model was selected as the optimal choice based on its superior balance between accuracy and simplicity, as evidenced by cross-validation results.

To further ensure generalization capability and avoid overfitting, we employed 10-fold cross-validation, partitioning the dataset into 10 equal subsets. In each iteration, nine subsets were used for training and one for testing, and the resulting performance metrics were averaged across all folds. This provided a robust estimate of the model’s predictive ability across all biometric parameters.

## 4. Experimental Results

**Figure 3:**
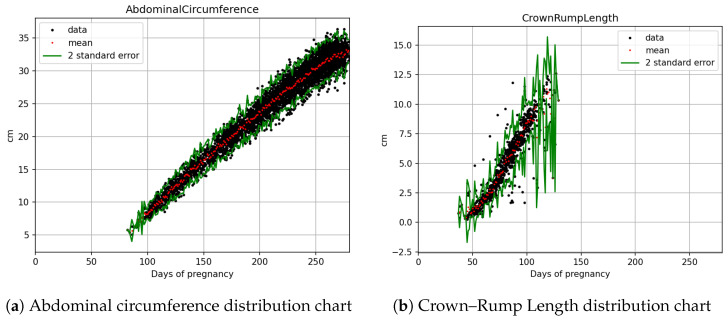
Fetal biometric distribution charts: (**a**) abdominal circumference and (**b**) crown–rump length, plotted against days of pregnancy. Black dots indicate data, red dots indicate mean, and green lines represent ±2 standard error.

**Figure 4:**
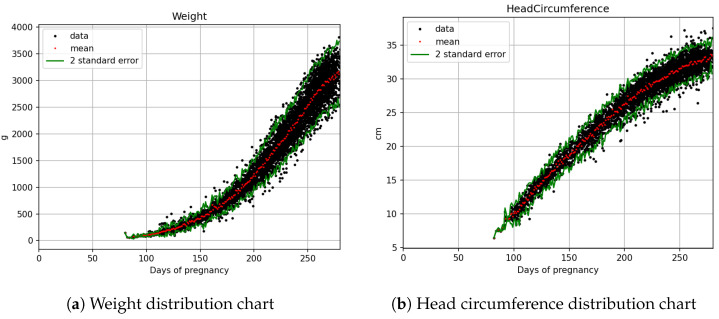
Fetal biometric distribution charts: (**a**) weight and (**b**) head circumference, with the mean and ±2 standard error.

**Figure 5:**
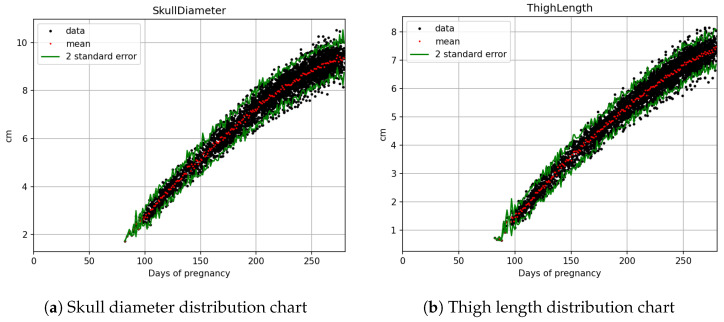
Fetal biometric distribution charts with fitted mean and ±2 standard error: (**a**) skull diameter and (**b**) thigh length.

The statistical count and splitting quantities of each field are as shown in Table 2. Based on Figure 3a,b, Figure 4a,b, and Figure 5a,b, it is evident that fetal abdominal circumference, fetal weight, head circumference, skull diameter, and thigh length exhibit a quadratic growth pattern with respect to gestational age.

We conducted curve fitting separately using Linear, Quadratic, and Cubic equations, and the results are shown in Table 3. From the table, it can be observed that fetal abdominal circumference, fetal weight, head circumference, skull diameter, and thigh length exhibit significantly higher $R^{2}$ values when fitted with Quadratic equations compared to Linear equations. The difference in $R^{2}$ values between Quadratic and Cubic equations is minimal. Considering computational efficiency and the improvement in $R^{2}$ values, we opted to employ Quadratic equations for the curve fitting.

**Figure 6:**
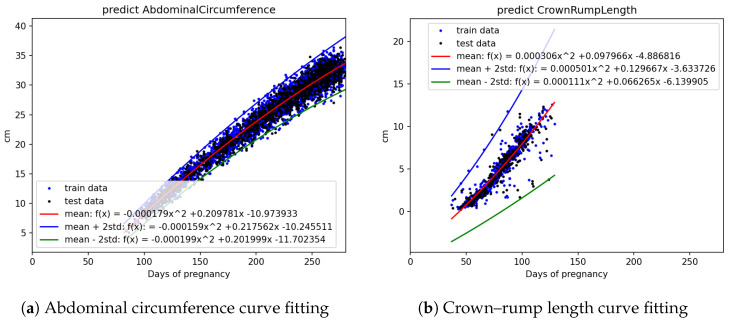
Curve fitting results for fetal biometric features using quadratic regression with ±2 standard deviation bounds: (**a**) abdominal circumference and (**b**) Crown–Rump Length.

**Figure 7:**
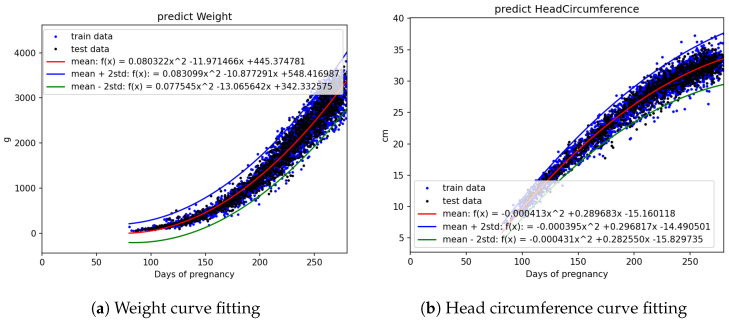
Curve fitting results using quadratic models with ±2 standard deviation intervals: (**a**) weight and (**b**) head circumference. Blue and black dots represent training and testing data, respectively.

**Figure 8:**
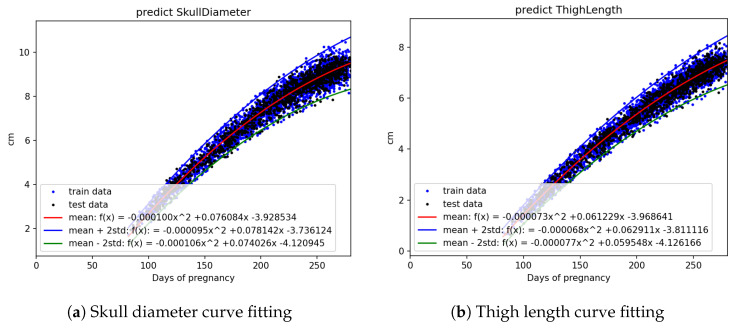
Curve fitting results using quadratic models with ±2 standard deviation bounds: (**a**) skull diameter and (**b**) thigh length. Training and testing datasets are marked in blue and black, respectively.

The equation obtained through Quadratic curve fitting is presented in Table 4, while the standard deviations of the parameters in the equation are shown in Table 5. By adding and subtracting two times the standard deviation of the parameters from the equation in Table 4, a confidence interval is derived. This confidence interval is illustrated in Figure 6, Figure 7 and Figure 8. It can be applied to the web interface so that when new values are entered, if they fall outside this interval, it is considered as an anomaly.

**Figure 9:**
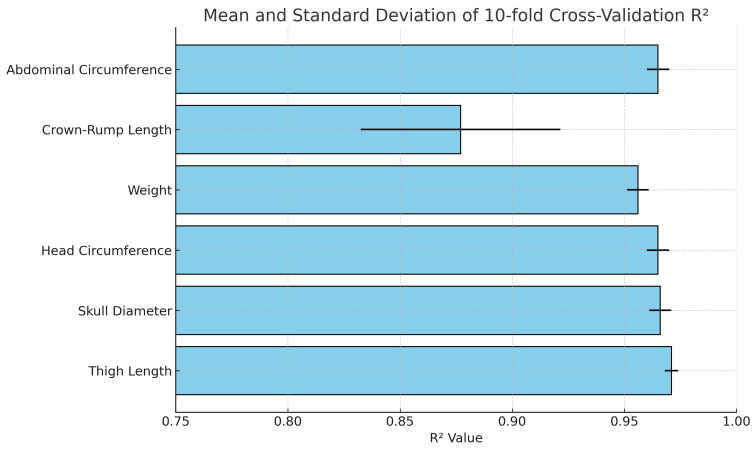
Mean and standard deviation of 10-fold cross-validation R^2^ values for each fetal biometric parameter using quadratic regression. The error bars represent the standard deviation across the 10 folds. Parameters such as femur length and head circumference show high reliability with both high mean R^2^ and low variance, whereas crown–rump length displays more variability, indicating relatively less stable predictive performance.

Furthermore, a validation process was conducted using 10-fold cross-validation, and the results are displayed in Table 6 and Figure 9. The 10-fold cross-validation consistently yielded high $R^{2}$ values, ranging from 0.88 to 0.97 across different iterations. These results reflect the robustness and reliability of our model. The high and consistent $R^{2}$ scores obtained from the cross-validation demonstrate the model’s strong predictive performance in capturing the relationship between the variables under study. This high level of consistency and accuracy further validates the model’s effectiveness in explaining the data.

**Figure 10:**
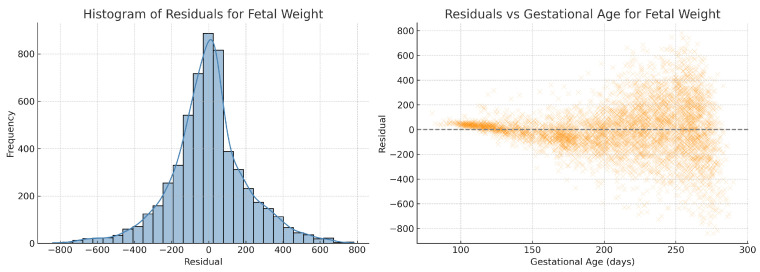
Residual analysis of the quadratic ridge regression model for fetal weight. (**Left**) Histogram of residuals showing an approximately normal distribution. (**Right**) Residuals plotted against gestational age, demonstrating no apparent heteroscedasticity across the range of gestational ages.

To further validate model assumptions, we performed residual analysis on the fitted quadratic regression for estimated fetal weight. As shown in Figure 10, the residuals are approximately normally distributed and exhibit no obvious heteroscedasticity across gestational age, supporting the appropriateness of the regression model. To assess regression assumptions across all biometric parameters, we performed residual analysis for abdominal circumference, fetal weight, head circumference, and femur length. All residual histograms demonstrated approximate normality, and residuals vs. gestational age plots did not reveal heteroscedasticity. Residual diagnostics for crown–rump length and skull diameter were also conducted but are not shown here due to similar characteristics.

### 4.1. Real Data Distribution

The black dots in Figure 3a represent the actual abdominal circumference data collected during prenatal examinations, with the y-axis representing length in centimeters and the x-axis representing gestational age in days. The red dots represent the average value of all the black dots on that day, while the green dots represent the average value plus and minus two standard deviations.

### 4.2. Curve Fitting Result

Using all the abdominal circumference data, divided into train and test sets represented by the blue and black dots in Figure 6a, curve fitting was performed on the train data to obtain the mean equation, mean +2 standard deviation equation, and mean −2 standard deviation equation. These three equations, represented by the red, blue, and green lines, respectively, were plotted on Figure 6a. The other parameters, including crown–rump length, weight, head circumference, skull diameter, and thigh length, were also analyzed using the same approach.

### 4.3. Real-Time Detection of Anomalies on Web Pages

**Figure 11:**
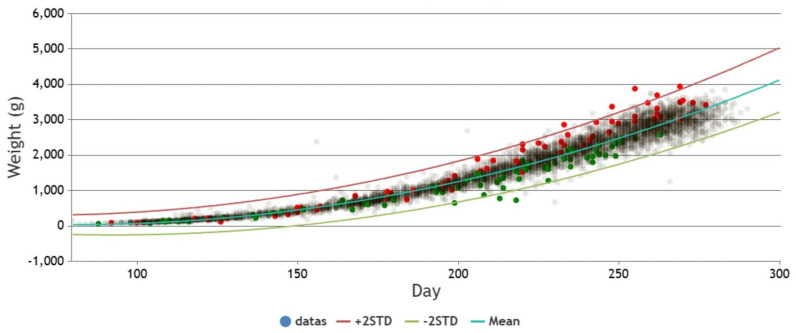
Relationship between Prenatal weight and fetal characteristics with gestational diabetes and preeclampsia.

The following Figure 11 is an actual example used on a webpage. It utilizes curve fitting to derive the mean equation and generates confidence intervals by incorporating two standard deviations. The prenatal weight data from the prenatal examinations are plotted on the graph. Pregnancies with gestational diabetes are marked as red dots, pregnancies with preeclampsia are marked as green dots, and others are marked as light gray. It can be observed from Figure 11 that pregnancies with gestational diabetes generally exhibit heavier fetal weights, while fetuses with maternal preeclampsia tend to have lighter fetal weights.

To statistically validate these visual trends, we performed a quantitative analysis of fetal weight residuals—that is, the difference between observed fetal weight and the model-predicted mean value. We categorized the data into three groups: (1) pregnancies with gestational diabetes, (2) pregnancies with preeclampsia, and (3) all others (control group).

A Kruskal–Wallis H test revealed a statistically significant difference among the three groups (p<0.001). Follow-up pairwise comparisons using Dunn’s test showed that:

- The gestational diabetes group had significantly higher residuals compared to the control group (p<0.01), suggesting fetal overgrowth.
- The preeclampsia group had significantly lower residuals than the control group (p<0.01), consistent with fetal growth restriction.

These statistical results confirm the observed patterns in Figure 11, providing further evidence of the clinical relevance of our anomaly detection framework.

## 5. Discussion

The utilization of prenatal ultrasound data and the application of curve fitting have helped us establish the fetal growth references of several parameters checked during the prenatal ultrasound examination with an excellent coefficient of determination (0.96–0.97), including abdominal circumference, crown–rump length, head circumference, skull diameter, thigh length, and body weight. The curve fitting model also provided valuable insights into the relationship between fetal growth and gestational age.

Measurements of crown–rump length face greater challenges in ultrasound imaging due to the initial short length of the fetus in early pregnancy, making it less accessible for accurate measurement and resulting in greater variations in the recorded measurements. Additionally, the curvature of the fetus in later stages of pregnancy poses difficulties in measurement, leading to a smaller amount of data collected. The combination of these factors results in the notably higher standard deviations observed in Figure 3b and Figure 6b.

Although several fetal growth curves have been proposed for clinical practice [18,19,20], it is possible to enhance the effectiveness of detecting abnormalities in fetal growth by using a chart that is tailored to the specific living environment, lifestyle, and genetic background of a local population. However, collecting data is time-consuming and labor-intensive. With the web-based data collection, we could easily and continuously obtain the prenatal ultrasound examination results and update the growth curve reference for clinical use. Fetal ultrasound measurements performed during pregnancy serve as the primary indicators of intrauterine growth quality. By comparing these measurements with standard fetal size charts, we can determine whether the fetus has a size that is appropriate for its gestational age [23]. When ultrasound measurements fall below the 10th percentile on the fetal size curve, it signifies a small-for-gestational age status. This condition is considered an important diagnostic criterion for fetal growth restriction, which is associated with an increased risk of adverse perinatal outcomes [21,24,25,26]. In our current study, the derived quadratic equation could be served as a predictive tool, enabling the identification of abnormal values and the early detection of growth retardation. By incorporating the quadratic equation into the data collection webpage, users can receive instant feedback on the abnormality of their input values. This real-time assessment helps in identifying potential issues and prompts users to seek appropriate medical attention. Furthermore, the data collection webpage plays a crucial role in presenting the distribution of all the collected data through charts. This visual representation allows data maintainers and researchers to quickly observe the patterns and trends in the dataset. Additionally, the drawn confidence intervals provide a means to identify data points that may deviate from the expected range, helping maintainers prioritize their attention and further investigate potential abnormalities.

The implementation of a webpage for data collection has proven to be an effective method for providing researchers with the latest data for analysis. By collecting data on prenatal examination, valuable insights into fetal growth and its relationship with gestational age have been obtained. Moving forward, there are opportunities to further enhance the analysis by integrating additional datasets. For example, combining the collected prenatal checkup data with air pollution data can provide insights into the potential impact of environmental factors on fetal development. Similarly, incorporating blood sampling data can offer a more comprehensive understanding of the physiological aspects related to prenatal health. By expanding the scope of analysis to include multiple data sources, researchers can gain a more holistic view of the factors influencing fetal growth and pregnancy outcomes. This integrated approach can lead to a deeper understanding of the complexities involved and contribute to the development of improved healthcare practices and interventions. Overall, the combination of the derived quadratic equation, automatic abnormality detection, and visual representation of data through charts and confidence intervals enhances the efficiency and effectiveness of monitoring fetal growth during pregnancy. This approach facilitates early intervention and timely medical support, leading to improved outcomes for both mothers and babies. In future work, we plan to explore incorporating maternal demographic and clinical features (e.g., BMI, age, and comorbidities) into the predictive model to enhance personalized risk assessment while preserving the model’s transparency and clinical applicability.

## 6. Conclusions

In summary, this project developed a website for collecting pregnancy-to-birth data to establish a local fetal growth reference. Using curve fitting and machine learning techniques, we gained valuable insights into fetal growth patterns and their association with gestational age. The Quadratic equations provided a robust model with high $R^{2}$ values for various prenatal measures, demonstrating effectiveness in capturing fetal growth dynamics.

However, measuring crown–rump length faced challenges, leading to higher variations and standard deviations. Although the webpage is efficient and user-friendly, data collection remains labor-intensive and time-consuming.

Future improvements could include integrating additional datasets, such as environmental and blood sampling data, to enhance understanding of prenatal health and fetal development. Continuous updates of the growth reference curve based on real-time data will further improve its clinical relevance and accuracy.

In conclusion, the data collection webpage is a valuable resource for researchers, enabling the development of tailored fetal growth curves. Leveraging technology and innovative methods advances prenatal care and improves health outcomes for pregnant women and their babies.

Future research could also explore several key directions to further enhance the clinical applicability and scientific value of the proposed system. First, longitudinal modeling techniques could be developed to account for sequential dependencies in fetal growth trajectories, enabling personalized predictions based on repeated measurements from the same fetus. Second, although this study emphasizes interpretability through polynomial regression, future work will include comparative evaluation with other explainable machine learning models—such as decision trees, rule-based learners, or generalized additive models—to assess trade-offs in performance, complexity, and deployability. Third, we plan to incorporate additional features such as maternal demographic data (e.g., age, BMI, and comorbidities), environmental exposure metrics (e.g., air pollution), and potentially genetic markers to construct more holistic and robust predictive models. Lastly, comparative validation using international databases such as TriNetX may help assess how fetal growth trends in the Taiwanese population align with or diverge from those observed globally.

## Figures and Tables

**Table 1:** Definition of biometric parameters for curve fitting.

Symbol	Parameter	Description
$x_{i}\in R$	Gestational Age (in days)	Independent variable representing fetal age at time of measurement
$y_{i}^{\left(\mathrm{AC}\right)}$	Abdominal Circumference (AC)	Reflects liver size and fat deposition; critical for estimating fetal weight and detecting IUGR
$y_{i}^{\left(\mathrm{CRL}\right)}$	Crown–Rump Length (CRL)	Most accurate marker for estimating gestational age in the first trimester
$y_{i}^{\left(\mathrm{EFW}\right)}$	Estimated Fetal Weight (EFW)	Central indicator for assessing fetal growth trajectory and identifying macrosomia
$y_{i}^{\left(\mathrm{HC}\right)}$	Head Circumference (HC)	Reflects cranial and neurological development; used to assess microcephaly or hydrocephalus
$y_{i}^{\left(\mathrm{BPD}\right)}$	Biparietal Diameter (BPD)	Standard fetal brain development metric, often used alongside HC
$y_{i}^{\left(\mathrm{FL}\right)}$	Femur Length (FL)	Represents long bone development; supplementary marker for fetal age and weight estimation

**Table 2:** Prenatal checkup data information.

Field	Number of Data	Number of Training Data	Number of Test Data
Abdominal Circumference	4594	3215	1379
Crown–Rump Length	985	689	296
Weight	5639	3947	1692
Head Circumference	4495	3146	1349
Skull Diameter	4573	3201	1372
Thigh Length	4531	3171	1360

**Table 3:** The $R^{2}$ values for curve fitting results using linear, quadratic, and cubic equations.

Field	Linear $R^{2}$	Quadratic $R^{2}$	Cubic $R^{2}$
Abdominal Circumference	0.9617	0.9650	0.9653
Crown–Rump Length	0.7937	0.7876	0.8077
Weight	0.9132	0.9539	0.9551
Head Circumference	0.9482	0.9676	0.9682
Skull Diameter	0.9549	0.9682	0.9686
Thigh Length	0.9640	0.9732	0.9732

**Table 4:** Quadratic equations of curve fitting for each fetal biometric parameter and their corresponding $R^{2}$ values.

Field	Equation	$R^{2}$
Abdominal Circumference	$f\left(x\right)=-0.000179x^{2}+0.209781x-10.973933$	0.965010
Crown–Rump Length	$f\left(x\right)=0.000306x^{2}+0.097966x-4.886816$	0.787560
Weight	$f\left(x\right)=0.080322x^{2}-11.971466x+445.374781$	0.953916
Head Circumference	$f\left(x\right)=-0.000413x^{2}+0.289683x-15.160118$	0.967605
Skull Diameter	$f\left(x\right)=-0.000100x^{2}+0.076084x-3.928534$	0.968174
Thigh Length	$f\left(x\right)=-0.000073x^{2}+0.061229x-3.968641$	0.973245

**Table 5:** Standard deviation errors for the parameters *a*, *b*, and *c* in the quadratic regression function $f\left(x\right)=ax^{2}+bx+c$.

Field	a	b	c
Abdominal Circumference	0.000010	0.003891	0.364211
Crown–Rump Length	0.000097	0.015851	0.626545
Weight	0.001388	0.547088	51.521103
Head Circumference	0.000009	0.003567	0.334808
Skull Diameter	0.000003	0.001029	0.096205
Thigh Length	0.000002	0.000841	0.078762

**Table 6:** $R^{2}$ values for quadratic curve fitting using 10-fold cross-validation for each fetal biometric parameter.

Field	1	2	3	4	5	6	7	8	9	10	AVG
Abdominal Circumference	0.97	0.96	0.96	0.97	0.96	0.97	0.96	0.96	0.97	0.97	0.96
Crown–Rump Length	0.93	0.87	0.83	0.91	0.86	0.90	0.84	0.79	0.91	0.93	0.88
Weight	0.96	0.96	0.96	0.95	0.95	0.96	0.95	0.95	0.96	0.96	0.96
Head Circumference	0.97	0.97	0.96	0.97	0.97	0.96	0.97	0.96	0.96	0.96	0.97
Skull Diameter	0.96	0.96	0.96	0.97	0.97	0.97	0.97	0.96	0.97	0.97	0.96
Thigh Length	0.97	0.98	0.97	0.97	0.97	0.97	0.97	0.97	0.97	0.97	0.97

## Data Availability

The original contributions presented in this study are included in the article. Further inquiries can be directed to the corresponding author.
